# Farm animal exposure setting impacts hemolytic uremic syndrome risk among Shiga toxin-producing *Escherichia coli* cases: Minnesota, 2010–2019

**DOI:** 10.1017/S0950268824000773

**Published:** 2024-05-16

**Authors:** Madhura S. Vachon, Joshua Rounds, Kirk Smith, Carlota Medus, Craig W. Hedberg, Carrie Klumb, Gillian A. M. Tarr

**Affiliations:** 1 Division of Environmental Health Sciences, University of Minnesota School of Public Health, Minneapolis, MN, USA; 2 Foodborne, Waterborne, Vectorborne, and Zoonotic Diseases Section, Minnesota Department of Health, Saint Paul, MN, USA

**Keywords:** Shiga toxin-producing *E. coli*, Hemolytic Uremic Syndrome, Ruminant Animal Contact

## Abstract

Shiga toxin-producing *Escherichia coli* (STEC) transmission occurs in ruminant contact settings and can lead to post-diarrheal hemolytic uremic syndrome (HUS). We investigated whether exposure setting (ruminant exposure from living or working on a farm, visiting a farm or animal contact venue, or both) influenced HUS development among individuals with laboratory-confirmed STEC infections using Minnesota surveillance data from 2010 to 2019. Logistic regression was performed to determine whether exposure setting was associated with HUS independent of age, gender, *stx2* gene detection, and county ruminants per capita. Among confirmed STEC cases, ruminant exposure only from living or working on a farm was not significantly associated with HUS compared to cases without any ruminant exposure (OR: 1.25; 95% CI: 0.51, 3.04). However, ruminant exposure only from visiting a farm or public animal contact venue was associated with HUS (OR: 2.53; 95% CI: 1.50, 4.24). Exposure from both settings was also associated with HUS (OR: 3.71; 95% CI: 1.39, 9.90). Exposure to ruminants when visiting farms or animal contact venues is an important predictor of HUS, even among people who live or work on farms with ruminants. All people, regardless of routine ruminant exposure, should take care in settings with ruminants to avoid infection with STEC.

## Introduction

Shiga toxin-producing *Escherichia coli* (STEC) transmission can occur at animal contact venues, which include agricultural fairs, petting zoos, and farm tours [1]. Ruminant animals, including cattle, sheep, and goats, are natural reservoirs of STEC [2]. Direct and indirect contact with these ruminants can increase the risk of STEC infection in humans [3, 4]. From 2009 to 2018, there were 64 reported STEC outbreaks associated with animal contact in the United States, resulting in 618 illnesses and 125 hospitalizations [5]. Infection with STEC can lead to the development of post-diarrheal hemolytic uremic syndrome (HUS), which is characterized by a triad of microangiopathic hemolytic anemia, thrombocytopenia, and acute renal injury. Progression to HUS is especially evident in younger age groups and among cases exposed to STEC strains that carry Shiga toxin 2 (Stx2), particularly when encoded by *stx2a* or *stx2d* genes [6].

A previous study identified an association between farm animal contact and progression to HUS among STEC cases in Indiana [7]. This association, which was independent of known risk factors for HUS (age, infection with an STEC strain that possesses *stx2*), indicates that the source of exposure could have implications for virulence [7]. Although earlier studies suggest that routine exposure to domesticated animals through living or working on a farm confers acquired immunity to STEC and its associated toxins, it is unknown whether HUS risk among STEC cases varies by the extent of prior exposure to farm animals [8, 9].

In this study, we aimed to determine, using surveillance data from the Minnesota Department of Health (MDH), whether ruminant exposure setting influences HUS risk.

## Methods

### Data collection and inclusion criteria

Laboratory-confirmed STEC cases reported to MDH from 2010 to 2019 were reviewed for analysis. STEC infection is required to be reported to MDH, and a clinical specimen or bacterial isolate must be submitted to the MDH Public Health Laboratory [10]. Latex agglutination or O antigen gene detection was used to conduct serotyping. Standardized polymerase chain reaction assay was used to determine s*tx* gene profiles.

STEC cases were deemed confirmed based on the Council of State and Territorial Epidemiologists case definitions associated with the year of disease notification. Evidence of confirmation included either isolation of *E. coli* O157:H7 or non-O157 strains accompanied by either *stx* gene detection or evidence of Shiga toxin production [11]. Among cases with confirmed STEC, HUS case classification was in accordance with the national surveillance case definition, which mandates acute illness diagnosed as HUS or thrombotic thrombocytopenic purpura accompanied by anemia and renal injury [12]. HUS is reportable to MDH immediately upon diagnosis [13]. We restricted this analysis to cases who tested positive for either *stx1* and *stx2* bacterial genes or *stx2* only, given that HUS is primarily associated with Stx2-producing strains [14, 15].

As part of routine surveillance activities, all STEC cases were interviewed with a standard case investigation questionnaire. Cases were asked whether they lived on, worked on, or visited a farm in the 7 days prior to illness onset, or visited a petting zoo, educational exhibit, fair, or other venue with animals in the week prior to illness. Those responding ‘yes’ to any of the above were asked about contact with specific animals (e.g., cattle, goats, sheep), including an ‘other’ category (Supplementary Material).

### Statistical analysis

The primary outcome of interest was HUS development, a binary categorical variable. Because HUS risk among people who lived, worked, or visited a farm without ruminants (3.3%) was similar to HUS risk among people who did not live, work, or visit a farm (4.1%), we classified our primary exposure variable as follows: (1) cases without any ruminant animal exposure; (2) cases whose only exposure to ruminants was because they lived or worked on a farm with ruminants; (3) cases whose only exposure to ruminants was because they visited a farm or animal contact venue with ruminants; and (4) cases who had exposure to ruminants because they both lived or worked on a farm with ruminants AND visited a farm or animal contact venue with ruminants. Visiting a venue did not distinguish between visiting a private farm and a public animal contact venue. Public animal contact venues in Minnesota include travelling petting zoos, pumpkin patches and corn mazes with farm animals, zoos with barnyard exhibits, agritourism farms, goat yoga, indoor petting zoos, and county and state fairs. Ruminant exposure was defined as direct contact with a ruminant or contact with a ruminant animal’s environment.

A descriptive analysis of the data was performed to determine the distribution of cases by STEC serogroup, detection of *stx* genes, age group, gender, and exposure setting. We also examined the distribution of ruminants per capita in each county [16–18]. Ruminants per capita were generated using cattle, sheep, and goat inventory from the United States Department of Agriculture (USDA) 2017 Census of Agriculture and population estimates from the Minnesota State Demographic Center [19, 20]. For continuous outcomes, bivariate comparisons were made using a two sample *t*-test for binary predictors and one-way analysis of variance (ANOVA) for categorical predictors with three or more categories. For binary outcomes, bivariate comparisons were made using a chi-squared test for binary categorical predictors.

We performed multiple imputations by chained equations to handle missing data using the R package ‘mice’ (Supplementary Methods) [21]. We confirmed the relationship between any ruminant exposure and progression to HUS by fitting a logistic regression on each of the imputed datasets, adjusting for age and *stx* profile, and pooled the results (Supplementary Material). For our primary analysis, we fit a logistic regression on each of the imputed datasets with HUS development as the dependent variable and exposure setting as an independent variable adjusted for age, gender, *stx* profile of the STEC strain, and county ruminants per capita. We conducted a sensitivity analysis to compare model estimates using STEC O157 cases only to all serogroups. Estimates were not vastly different; thus all serogroups were included in our final model. Results were pooled across datasets. We examined the interaction between age and exposure setting and used a likelihood ratio test to assess the change in residual deviance between the full and reduced model. The interaction term was dropped from our final model after it was determined that the difference between the two models was not significant. Regression coefficients were exponentiated to obtain odds ratios (ORs), and 95% confidence intervals (CIs) were calculated from pooled standard errors obtained using Rubin’s rules [22].

## Results

From 2010 to 2019 in Minnesota, there were 1 660 STEC-confirmed cases with strains that tested positive for either *stx1* and *stx2* or *stx2* only. Of these, 377 (23%) were aged 5 years or under. The majority of cases (1 147; 69%) tested positive for STEC O157. In total, 103 cases (6%) developed HUS. Of children aged 5 years or under, 58 (15%) developed HUS (Table 1). There was a significant difference in mean county ruminants per capita by exposure setting (*F* = 9.96, *p* < 0.0001). Mean county ruminants per capita were significantly higher in counties where cases with ruminant exposure lived or worked on a farm compared to cases with no ruminant exposure (*p* < 0.0001). There was a significant association between cases who tested positive for *stx2* only and HUS development compared to cases who tested positive for both *stx1* and *stx2* (Chi-square = 18.2, *p* < 0.0001).

**Table 1.** Descriptive summary of laboratory-confirmed Shiga toxin-producing *Escherichia coli* cases by exposure setting, age group, serogroup, Shiga toxin gene (*stx*) profile, county ruminant per capita, and hemolytic uremic syndrome (HUS) status – Minnesota, 2010–2019

	Cases without any ruminant animal exposure				Cases whose only exposure to ruminants was because they lived or worked on a farm with ruminants				Cases who had exposure to ruminants because they both lived or worked on a farm with ruminants AND visited a farm or other animal venue with ruminants				Cases whose only exposure to ruminants was because they visited a farm or other animal venue with ruminants			
Total	*n*	%	HUS	% HUS	*n*	%	HUS	% HUS	*n*	%	HUS	% HUS	*n*	%	HUS	% HUS
	1 350	81.3	67	5.0	88	5.3	6	6.8	28	1.7	6	21.4	194	11.7	24	12.4
Age Group																
≤5 years	280	20.7^a^	35	12.5^b^	24	27.3	4	16.7	10	35.7	5	50.0	63	32.5	14	22.2
6–10 years	104	7.7	11	10.6	5	5.7	1	20.0	4	14.3	1	25.0	31	16.0	5	16.1
11–18 years	189	14.0	4	2.1	13	14.8	0	0.0	9	32.1	0	0.0	36	18.6	2	5.6
19–45 years	406	30.1	5	1.2	18	20.5	0	0.0	2	7.1	0	0.0	49	25.3	2	4.1
46–65 years	198	14.7	3	1.5	23	26.1	1	4.3	1	3.6	0	0	6	3.1	0	0.0
65+ years	173	12.8	9	5.2	5	5.7	0	0.0	2	7.1	0	0	9	4.6	1	11.1
Gender																
Male	595	44.1	23	3.9	36	40.9	3	8.3	10	35.7	1	10.0	84	43.3	8	9.5
Female	754	55.9	44	5.8	52	59.1	3	5.8	18	64.3	5	27.8	110	56.7	16	14.5
Serogroup																
O157	928	77.5	63	6.8	59	71.1	5	8.5	21	77.8	3	14.3	139	77.2	20	14.4
O103	19	1.6	0	0.0	1	1.2	0	0.0	0	0.0	0	–	3	1.7	0	0.0
O26	26	2.2	0	0.0	1	1.2	0	0.0	0	0.0	0	–	2	1.1	0	0.0
O111	67	5.6	2	3.0	7	8.4	0	0.0	1	3.7	0	0.0	17	9.4	3	17.6
O145	60	5.0	0	0.0	4	4.8	0	0.0	4	14.8	2	50.0	8	4.4	1	12.5
O121	66	5.5	0	0.0	8	9.6	0	0.0	0	0.0	0	–	7	3.9	0	0.0
O45	5	0.4	0	0.0	0	0.0	0	–	0	0.0	0	–	0	0.0	0	–
Other	27	2.3	1	3.7	3	3.6	0	0.0	1	3.7	0	0.0	4	2.2	0	0.0
*stx* Profile																
*stx1* and *stx2*	608	45.0	17	2.8	35	39.8	2	5.7	11	39.3	0	0.0	118	60.8	8	6.8
*stx2*	742	55.0	50	6.7	53	60.2	4	7.5	17	60.7	6	35.3	76	39.2	16	21.1
County Ruminant per Capita	Med.	IQR	Med.	IQR	Med.	IQR	Med.	IQR	Med.	IQR	Med.	IQR	Med.	IQR	Med.	IQR
	0.21	0.86	0.24	1.17	1.3 = 38	1.69	1.05	0.66	0.80	1.22	0.25	0.92	0.38	1.22	0.27	0.93

Abbreviations: HUS, hemolytic uremic syndrome; Med., median; *stx*, Shiga toxin bacterial gene.

a Column percentage is taken to determine case distribution by age group.

b Row percentage is taken to determine %HUS by age group.

In our sample, 1 350 cases (81%) did not report any ruminant exposure, 88 (5%) only had exposure to ruminants because they lived or worked on a farm with ruminants, 194 (12%) only had exposure to ruminants because they visited a farm or other animal venue with ruminants, and 28 (1.7%) both lived or worked on a farm with ruminants AND visited a farm or other animal venue with ruminants (Table 1). In our final adjusted model, ruminant exposure only from living or working on a farm was not significantly associated with HUS compared to STEC cases without any ruminant contact or exposure (OR: 1.25; 95% CI: 0.51, 3.04). Conversely, having ruminant exposure only from visiting a farm or other venue was associated with HUS (OR: 2.53; 95% CI: 1.50, 4.24). Ruminant exposure from both visiting a farm or other animal venue AND living or working on a farm was also associated with HUS (OR: 3.71; 95% CI: 1.39, 9.90). Relative to strains positive for both *stx1* and *stx2*, strains positive for only *stx2* were significantly associated with HUS (OR: 3.04; 95% CI: 1.91, 4.83). As expected, younger age was associated with HUS development (OR: 0.97; 95% CI: 0.96, 0.98). Female gender was also linked to HUS development (OR: 0.54; 95% CI: 0.35, 0.83). County ruminant per capita was not associated with HUS in the final model (OR: 0.97; 95% CI: 0.84, 1.12) (Table 2).

**Table 2.** Association between exposure setting and hemolytic uremic syndrome (HUS) adjusted for gender, age per year of life, Shiga toxin gene (*stx*) profile, and county ruminant per capita – Minnesota, 2010–2019

	OR	95% CI	
HUS		LCI	UCI
Exposure setting			
*(Reference: No ruminant contact or exposure)*			
Live or work on a farm with ruminants only	1.25	0.51	3.04
Both live or work on a farm with ruminants AND Visit a farm or other animal venue with ruminants	3.71	1.39	9.90
Visit a farm or other animal venue with ruminants only	2.53	1.50	4.24
Gender			
* (Reference: Female)*			
Male	0.54	0.35	0.83
*stx* Profile			
*(Reference: stx1* and *stx2)*			
*stx2*	3.04	1.91	4.83
Age per year of life	0.97	0.96	0.98
County ruminant per capita	0.97	0.84	1.12

## Discussion

Our findings demonstrate that visiting a farm or other animal venue significantly increases the risk of HUS among individuals infected with STEC, with the magnitude of the risk differing somewhat based on whether they also had contact with ruminants at home or work. This is independent of traditional risk factors for HUS, including age and presence of *stx2.*

While several studies have established an increased risk of STEC infection due to direct ruminant contact [23, 24], living in a ruminant-dense area [16–18], and visiting farms or petting zoos [25–28], whether ruminant exposure is also associated with increased risk of HUS among individuals with STEC infections is less clear. More recent evidence indicated that the HUS rate in animal contact STEC outbreaks (9%) was significantly higher than the HUS rate in STEC outbreaks with other modes of transmission (6%) [29]. Our findings corroborate findings from Indiana that ruminant animal exposure increases the risk of HUS development among people with STEC infection independent of known risk factors [7]. Specifically, HUS risk significantly increased among people who were exposed to ruminants while visiting a farm or other animal venue. Although county ruminants per capita have a large effect on STEC infection risk, it had no effect on our estimates of HUS risk from animal exposure. This could be a consequence of either specifically examining HUS risk or from accounting for direct exposure in our model.

There are several potential explanations for why exposure to ruminants is associated with an increased risk of progression to HUS among confirmed STEC cases. Stress associated with transportation and unfamiliar surroundings may cause ruminant animals to shed higher bacterial volumes at animal contact venues [30]. This would impact the exposure dose at such events. The commingling of a variety of animals also increases the diversity of bacterial strains contained in a single location [31]. STEC isolated from ruminants harbour known virulence factors that contribute to clinical severity [32]. Greater diversity of bacterial strains and virulence factors could also contribute to more severe disease manifestations among those infected with STEC at animal contact venues.

Our findings suggest that acquired immunity to home farm-specific STEC strains is not protective against other strains that may be present at animal contact venues, particularly among young children. We support this by showing that exposure to ruminants from both living or working on a farm AND visiting a farm or other public animal contact venue was associated with an increased HUS risk, with a higher odds ratio than that observed with visiting a farm or public animal contact venue only. However, all HUS cases in both categories were aged 10 or younger. This is consistent with evidence of acquired immunity to STEC and its associated toxins among adults who live or work on farms [8, 9], as acquired immunity is commonly not present yet in younger children who live on farms [4]. These findings are understandable given that, generally, adults have more developed immune systems than young children [33].

The results of this study have implications for individual prevention, clinical awareness, and public health intervention. Parents of young children should remain cautious in all exposure settings with live ruminant animals given that immune mechanisms from routine exposure to these animals may not protect against severe clinical outcomes from STEC. Healthcare providers treating young children or older adults for acute STEC infections should be aware of the increased risk of HUS among cases who visited an animal contact venue with ruminants. Venue operators should make the public aware that exposure to farm animals and livestock from animal contact venues places one at an increased risk of severe clinical consequences from infection, regardless of prior exposure or experience with animals. While there are many sources of STEC infections, and only 19% of cases in our study had ruminant contact, we have demonstrated that ruminant contact significantly increases the likelihood of infection progressing to HUS, with 35% of HUS cases reporting ruminant contact. Thus, measures to reduce infections through ruminant contact have the potential for an outsized impact on HUS burden.

This study was limited to STEC infections identified through pathogen-specific surveillance. Surveillance limitations, such as care-seeking biases, may impact the generalizability of our results. Inadequate sample size prevented us from examining non-linear relationships between age and HUS risk. The creation of four exposure-setting categories was necessary, despite the smaller number of HUS cases in each category, given the differences between them. However, since the number of events was low, particularly in categories where people lived or worked on a farm, model estimates were relatively imprecise. We were also unable to examine potential mediation by known virulence factors. Additionally, we could not examine the effect of exposure to different *stx* subtypes on HUS development given that subtyping information was not available for all isolates.

In addition to being a risk factor for STEC infection, exposure to ruminant animals could be an important predictor of HUS among individuals with STEC infection. Visiting a farm or other animal venue with ruminant animals may increase the likelihood of high-risk STEC exposure. All members of the public should take additional care at public animal contact venues to avoid infection from animal contact. This can be done by practicing more frequent handwashing, avoiding food consumption or other hand-to-mouth contact in animal areas, and limiting strollers and other inanimate objects in animal areas.

## Supporting information

Vachon et al. supplementary material 1 Vachon et al. supplementary material

Vachon et al. supplementary material 2 Vachon et al. supplementary material

## Data Availability

The data that support the findings of this study are available from the Minnesota Department of Health. Restrictions apply to the availability of these data.

## Supplementary material

The supplementary material for this article can be found at http://doi.org/10.1017/S0950268824000773.
