# Supplementary material for: Farm animal exposure setting impacts hemolytic uremic syndrome risk among Shiga toxin-producing Escherichia coli cases: Minnesota, 2010–2019
Source: Epidemiol Infect. 2024 May 16;152:e96. doi: 10.1017/S0950268824000773 (PMC11736442; doi:10.1017/S0950268824000773)
Supplement: Vachon et al. supplementary material 1 — Vachon et al. supplementary material [file S0950268824000773sup001.docx]

Date:___/___/___ Interviewed in: English Other: __________________

Interviewer:____________Bacteria________________Species/serotype__________________Subtype_______

**Enteric Disease Worksheet**

Patient’s Name (last, first)_______________________________________________ DOB: ___/___/___

Parent’s Name (if child)_________________________________________________

Symptom History

Vomiting Y N Chills Y N What was first symptom?__________________

Diarrhea Y N Headache Y N Date of onset: (mm/dd/yy) ___/___/___

Stools/24 hr _____ Muscle Aches Y N Time of onset: (military) ____________

Blood in stool Y N Joint Pain Y N Date of onset diarrhea: ___/___/___

Cramps Y N Time of onset of diarrhea: __________

Fever Y N Temp _____ Duration of diarrhea (days) _________

Comments: Other ____________ Date of recovery: ___/___/___

Time of recovery: __________

Did you visit an emergency room for this illness? Yes □ No □ If yes, how many times? ______

Did you visit an outpatient clinic for this illness? Yes □ No □ If yes, how many times? ______

(e.g., urgent care, primary care)

If hospitalized, did you stay in an Intensive Care Unit (ICU) Yes □ No □

or Critical Care Unit (CCU)?

Were you treated with any antibiotics after the onset of this illness? Yes □ No □ If yes:

| Antibiotic #1 ____________________  Date prescribed: ___/___/___  Start date ___/___/___  End date ___/___/___  Duration (days) ____________  Did you take the antibiotic before you submitted your stool culture?  Yes □ No □  If yes, how many days before  culture?______________  or SAME DAY □ | Antibiotic #2 ____________________  Date prescribed: ___/___/___  Start date ___/___/___  End date ___/___/___  Duration (days) ____________  Did you take the antibiotic before you submitted your stool culture?  Yes □ No □  If yes, how many days before  culture?______________  or SAME DAY □ | Antibiotic #3 ____________________  Date prescribed: ___/___/___  Start date ___/___/___  End date ___/___/___  Duration (days) ____________  Did you take the antibiotic before you submitted your stool culture?  Yes □ No □  If yes, how many days before  culture?______________  or SAME DAY □ |
| --- | --- | --- |

1. Did you travel anywhere **during the week prior** to your illness, including immigration/adoption? Yes □ No □

**If yes**, where? _______________________________________________ when ? ___/___/___ thru ___/___/___

If airline travel, what airline? ____________________________________________________

Where did you stay? (name of hotel/resort): ________________________________________________________

Did you travel internationally in the **6 months prior** to your illness? Yes □ No □

**If yes**, where? _______________________________________________ when? ___/___/___ thru ___/___/___

Did anyone in your household travel internationally in the 6 months prior to your illness? Yes □ No □

**If yes**, where? _______________________________________________ when? ___/___/___ thru ___/___/___

2. Did you drink water from any of the following sources during the week prior to your illness?

Well water [location (home, cabin); address] ___________________________________________ Yes □ No □

Bottled Water Brand: _______________________________________________ Yes □ No □

Other (such as from a stream while camping) Yes □ No □

- Specify_____________________________________________________________

3. Did you swim in an ocean, lake, river, pool or splash pad in the week before your illness? Yes □ No □

**If yes**, where? __________________________________________________when?______________

4. Where did you shop for groceries eaten during the week before your illness? (include grocery stores, warehouse

stores, online retailers, gas stations, etc.) ______________________________________________________

________________________________________________________________________________________

5. In the week prior, did you consume meat from any place other than the grocery store? (hunting, butcher

shop, private kill, slaughterhouse or place where you purchased a live animal for slaughter) Yes □ No □

**If yes**, type of meat? ______________________ Source:_______________________________________

Where processed? _____________________________________________________________________

6. Where and when did you get any milk you drank the week before your illness?

_________________________________________________________________________________

Brand and type (e.g., skim, 2%): _______________________________________________________

Was any of it unpasteurized? Yes □ No □

7. During the 7 days prior to your illness, did you live on, work on, or visit a farm? Yes □ No □

**If yes,** name, location, and dates at farm (other than home farm):

□ Live on farm

□ Work on farm: _______________________________________________________ When? ____________

□ Visit farm: ___________________________________________________________When? ____________

8. Did you visit a petting zoo, educational exhibit, fair or other venue with animals in the week before your illness?

Yes □ No □

Name and location of petting zoo/fair: ________________________________________ When? ___/___/___

9. **If yes to questions 7 or 8:** Were any of the following animals present? If yes, did you have any contact with them?

|  | **Home** | | | **Work** | | | **Other Farm** | | | **Petting Zoo/Other Venues** | | |
| --- | --- | --- | --- | --- | --- | --- | --- | --- | --- | --- | --- | --- |
|  | Present –  No Contact | Contact | Describe Contact | Present –  No Contact | Contact | Describe Contact | Present –  No Contact | Contact | Describe Contact | Present –  No Contact | Contact | Describe Contact |
| **Cow** | □ | □ |  | □ | □ |  | □ | □ |  | □ | □ |  |
| **Goat** | □ | □ |  | □ | □ |  | □ | □ |  | □ | □ |  |
| **Sheep** | □ | □ |  | □ | □ |  | □ | □ |  | □ | □ |  |
| **Pig** | □ | □ |  | □ | □ |  | □ | □ |  | □ | □ |  |
| **Chicken** | □ | □ |  | □ | □ |  | □ | □ |  | □ | □ |  |
| **Turkey** | □ | □ |  | □ | □ |  | □ | □ |  | □ | □ |  |
| **Other:** | □  □  □ | □  □  □ |  | □  □  □ | □  □  □ |  | □  □  □ | □  □  □ |  | □  □  □ | □  □  □ |  |

10. Did you have contact with any animal manure or composted animal manure? Yes □ No □

**If yes,** describe type contact (e.g., gardening): ___________________________________________________

11. During the week prior to your illness, did you have any contact with pets (e.g. dogs, cats, reptiles, hedgehogs, rodents, or pet livestock such as chickens, pigs, or horses) at home or elsewhere (including school or someone else’s home)? Yes □ No □

**If yes,** what kind of animal(s)? ______________________________________________________________

***If reptile exposure, complete reptile questionnaire.*** Questionnaire completed? Yes □ No □

**If yes,** Did you feed animal-based products such as rawhides, pig’s ears or cow hooves, treats or raw foods to

your pet? Yes ⬜ No ⬜

Type: ______________________________________________________________________________

12. Do you know of anyone else with a diarrheal illness prior to or following your illness? Yes ⬜ No ⬜

**If yes**, when were they ill? Date: __/___/___ Before ⬜ After ⬜ Same time ⬜

Who? _______________________________________

***If children are ill, ask about daycare.***

13. Did you attend or work at a daycare or preschool prior to or following your illness? Yes ⬜ No ⬜

**If yes**, what days: __________________________________________________________

Name of Daycare: _______________________________________________________________

Name of Daycare Director: _______________________________________________________

City: ____________________________

# Phone Number: ____________________

Are you aware of any other illness in daycare? Yes ⬜ No ⬜

Did you attend or work at a daycare with a diarrheal illness? Yes ⬜ No ⬜ Dates: ________________

**For ALL daycare attendees and employees:**

We will contact the daycare provider to determine if any other children have been ill and to provide information and recommendations to prevent the spread of illness. Do you have any concerns about disclosing your/your child’s name to the daycare? ⬜ Yes, I do have concerns ⬜ No, I do not have concerns

If you/your child still has diarrhea, you/he/she may not attend daycare until fully recovered. ⬜ **Tennessen read**

**For *E. coli* O157 or stx2 or stx pending/unknown daycare attendees and employees ONLY:**

Additionally, because Shiga-toxin producing *E. coli* can cause severe complications and can be easily spread at daycare, restrictions apply to children/adults with this type of illness who attend/work at daycare. An epidemiologist will contact you as soon as possible to discuss these restrictions. You/your child may not attend daycare at this time. ⬜ **Tennessen read**

14. Did you attend any large gatherings the week before your illness (weddings, receptions, showers, parties,

festivals, fairs, etc.)? Yes ⬜ No ⬜

**If yes**, when: ___/___/___

What type of event? _________________________________________________________

Where?___________________________________________________________________

Foods served? _____________________________________________________________

Others ill? Yes ⬜ No ⬜

**If yes,** describe: ____________________________________________________________

15. Did you eat any food or beverages from any restaurants, coffee shops, cafeterias, delis, food stands/street

vendors, or meal delivery services during the seven days before your illness? Yes ⬜ No ⬜

1. Name:_______________________________________ Date: ___/___/___ Time: ____________

Address: ______________________________________________________________________

foods eaten: ___________________________________________________________________

1. Name:_______________________________________ Date: ___/___/___ Time: ____________

Address: ______________________________________________________________________

foods eaten: ___________________________________________________________________

1. Name:_______________________________________ Date: ___/___/___ Time: ____________

Address: ______________________________________________________________________

foods eaten: ___________________________________________________________________

1. Name:_______________________________________ Date: ___/___/___ Time: ____________

Address: ______________________________________________________________________

foods eaten: ___________________________________________________________________

1. Name:_______________________________________ Date: ___/___/___ Time: ____________

Address: ______________________________________________________________________

foods eaten: ___________________________________________________________________

1. Name:_______________________________________ Date: ___/___/___ Time: ____________

Address: ______________________________________________________________________

foods eaten: ___________________________________________________________________

***Please prompt case with previously reported events and restaurants when asking about 5-day food history**

**Did you (your child) have:** Food Allergies? Yes ⬜ No ⬜ Describe:_______________

Vegetarian or vegan diet? Yes ⬜ No ⬜

Special or restricted diet? Yes ⬜ No ⬜ Describe:_______________

Date/day prior to onset

___/___/___

Ate at Ate outside Outside

**Time of Meal** Meal home of home location Foods eaten

__________ Breakfast _______________ __________________

__________ Lunch _______________ __________________

__________ Dinner _______________ __________________

__________ Other _______________ __________________

___/___/___

__________ Breakfast _______________ __________________

__________ Lunch _______________ __________________

__________ Dinner _______________ __________________

___________ Other ________________ ____________________

___/___/___

__________ Breakfast _______________ __________________

__________ Lunch _______________ __________________

__________ Dinner _______________ __________________

__________ Other _______________ __________________

___/___/___

__________ Breakfast _______________ __________________

__________ Lunch _______________ __________________

__________ Dinner _______________ __________________

__________ Other _______________ __________________

___/___/___

__________ Breakfast _______________ __________________

__________ Lunch _______________ __________________

__________ Dinner _______________ __________________

__________ Other _______________ __________________

### FOOD CONSUMPTION HISTORY

Please indicate for each of the food items listed below whether you definitely ate it, maybe ate it, or definitely did not eat it during the week before illness onset.

| **Item** | **Ate** | **Did not eat** | | **May have eaten** | | **How prepared** | **Variety or brand** | | **Date purchased** | | | **Grocery store where purchased** | | **Date eaten** | | **Restaurant where eaten**  **(include address)** | |
| --- | --- | --- | --- | --- | --- | --- | --- | --- | --- | --- | --- | --- | --- | --- | --- | --- | --- |
| **EGGS/DAIRY** |  | | | | | | | | | | | | | | | | |
| Eggs |  |  | |  | |  |  | | / / | | |  | | / / | |  | |
|  | a. Did you eat a prepared dish (e.g., egg bake, quiche, custard) that contained egg? Yes ⬜ No ⬜ Type of dish: ___________________  b. Did you sample any batter that used raw egg in the preparation e.g., cookie dough? Yes ⬜ No ⬜ | | | | | | | | | | | | | | | | |
| Did you or someone in your house use flour in any baking or cooking? Yes ⬜ No ⬜  If yes, what did you bake/cook ? ___________________________ Brand & Variety (i.e. bleached/unbleached) ____________________ Where purchased : ___________________________ | | | | | | | | | | | | | | | | | |
| Shredded cheese |  |  | |  | |  |  | | / / | | |  | | / / | |  | |
| Block cheese or cheese slices (specify) |  |  | |  | |  |  | | / / | | |  | | / / | |  | |
| String cheese or Cheese curds |  |  | |  | |  |  | | / / | | |  | | / / | |  | |
| Queso fresco or other Mexican style cheese |  |  | |  | |  |  | | / / | | |  | | / / | |  | |
| Other cheeses (e.g., Gouda, blue, feta, chevre, brie, goat, sheep) |  |  | |  | |  |  | | / / | | |  | | / / | |  | |
| Ice cream |  |  | |  | |  |  | | / / | | |  | | / / | |  | |
| Frozen dessert treats |  |  | |  | |  |  | | / / | | |  | | / / | |  | |
| Yogurt |  |  | |  | |  |  | | / / | | |  | | / / | |  | |
| Milk alternatives (e.g., soy, almond, or rice milk) |  |  | |  | |  |  | | / / | | |  | | / / | |  | |
| Other dairy (e.g., cottage cheese, cream cheese, sour cream) |  |  | |  | |  |  | | / / | | |  | | / / | |  | |
| **Item** | **Ate** | **Did not eat** | | **May have eaten** | **How prepared** | | **Variety or brand** | | | **Date purchased** | | **Grocery store where purchased** | | | **Date eaten** | **Restaurant where eaten**  **(include address)** | |
| **MEAT/**  **POULTRY** |  | | | | | | | | | | | | | | | | |
| Ground beef |  |  | |  |  | |  | | | / / | |  | | | / / |  | |
|  | a. Ground beef as an ingredient: type of dish ___________________________________________________  b. Ground beef: **raw** Y N U **rare (red in middle)** Y N U **medium (pink in middle)** Y N U **well done (no pink)** Y N U  c. How was it packaged? ⬜ Tube ⬜ Tray ⬜ Pre-made patties ⬜ Other :________________  d. Package size :_________________ Percent lean (e.g., 80/20) : _____________ | | | | | | | | | | | | | | | | |
| Veal |  |  | |  |  | |  | | | / / | |  | | | / / |  | |
| Other beef (e.g., steak) |  |  | |  |  | |  | | | / / | |  | | | / / |  | |
| Chicken (including ground) |  |  | |  |  | |  | | | / / | |  | | | / / |  | |
| Stuffed chicken product (e.g., chicken Kiev) |  |  | |  |  | |  | | | / / | |  | | | / / |  | |
| Turkey (including ground) |  |  | |  |  | |  | | | / / | |  | | | / / |  | |
| Pork (e.g., ham, bacon) |  |  | |  |  | |  | | | / / | |  | | | / / |  | |
| Lamb |  |  | |  |  | |  | | | / / | |  | | | / / |  | |
| Sausage |  |  | |  |  | |  | | | / / | |  | | | / / |  | |
| Venison or other meat/poultry |  |  | |  |  | |  | | | / / | |  | | | / / |  | |
| Fish (incl. sushi, or ceviche) |  |  | |  |  | |  | | | / / | |  | | | / / |  | |
| Shrimp |  |  | |  |  | |  | | | / / | |  | | | / / |  | |
| Other seafood |  |  | |  |  | |  | | | / / | |  | | | / / |  | |
| **Item** | **Ate** | **Did not eat** | | **May have eaten** | **How prepared** | | **Variety or brand** | | | **Date purchased** | | **Grocery store where purchased** | | | **Date eaten** | **Restaurant where eaten**  **(include address)** | |
| **FROZEN PROCESSED FOODS** |  | | | | | | | | | | | | | | | | |
| Frozen dinners/entrees (e.g., Lean Cuisine, pot pies) |  |  | |  |  | |  | | / / | | |  | | / / | |  | |
| Frozen pizza |  |  | |  |  | |  | | / / | | |  | | / / | |  | |
| Other frozen microwaveable foods |  |  | |  |  | |  | | / / | | |  | | / / | |  | |
| **FRUITS**  **(fresh, frozen, or dried)** |  | | | | | | | | | | | | | | | | |
| Oranges |  |  | |  |  | |  | | / / | | |  | | / / | |  | |
| Other citrus (e.g., grapefruit, lemon, lime, tangerine) |  |  | |  |  | |  | | / / | | |  | | / / | |  | |
| Pears |  |  | |  |  | |  | | / / | | |  | | / / | |  | |
| Apples |  |  | |  |  | |  | | / / | | |  | | / / | |  | |
| Other tree fruit  (e.g., apricot, plum, nectarine, peach) |  |  | |  |  | |  | | / / | | |  | | / / | |  | |
| Strawberries |  |  | |  |  | |  | | / / | | |  | | / / | |  | |
| Other berries (e.g., blue, black, or raspberries) |  |  | |  |  | |  | | / / | | |  | | / / | |  | |
| Grapes (specify color) |  |  | |  |  | |  | | / / | | |  | | / / | |  | |
| Bananas |  |  | |  |  | |  | | / / | | |  | | / / | |  | |
| Watermelon (specify seeds or seedless) |  |  | |  |  | |  | | / / | | |  | | / / | |  | |
| **Item**  (Fruits cont.) | **Ate** | **Did not eat** | **May have eaten** | | **How prepared** | | | **Variety or brand** | | | **Date purchased** | | **Grocery store where purchased** | | **Date eaten** | | **Restaurant where eaten**  **(include address)** |
| Cantaloupe |  |  |  | |  | | |  | | | / / | |  | | / / | |  |
| Honeydew or other melon |  |  |  | |  | | |  | | | / / | |  | | / / | |  |
| Other fruit (e.g., mango, pomegranate, kiwi, pineapple) |  |  |  | |  | | |  | | | / / | |  | | / / | |  |
| Fruit salad (specify ingredients, brand) |  |  |  | |  | | |  | | | / / | |  | | / / | |  |
| Unpasteurized apple  cider |  |  |  | |  | | |  | | | / / | |  | | / / | |  |
| Other unpasteurized juice (e.g., fresh-squeezed, or smoothies) |  |  |  | |  | | |  | | | / / | |  | | / / | |  |
| Other juice |  |  |  | |  | | |  | | | / / | |  | | / / | |  |
| **VEGETABLES**  **(fresh or frozen)** |  | | | | | | | | | | | | | | | | |
| Prepackaged salad (specify bag or clamshell) |  |  |  | |  | | |  | | | / / | |  | | / / | |  |
| Iceberg |  |  |  | |  | | |  | | | / / | |  | | / / | |  |
| Romaine |  |  |  | |  | | |  | | | / / | |  | | / / | |  |
| Spinach |  |  |  | |  | | |  | | | / / | |  | | / / | |  |
| Cabbage |  |  |  | |  | | |  | | | / / | |  | | / / | |  |
| Other lettuce/leafy greens (e.g., spring mix, kale, arugula, Swiss chard, endive) |  |  |  | |  | | |  | | | / / | |  | | / / | |  |
| Tomatoes (e.g., vine-on, heirloom, roma, beefsteak, grape, incl. on a sandwich/salad) |  |  |  | |  | | |  | | | / / | |  | | / / | |  |
| **Item**  (Vegetables cont.) | **Ate** | **Did not eat** | **May have eaten** | | **How prepared** | | | **Variety or brand** | | | **Date purchased** | | **Grocery store where purchased** | | **Date eaten** | | **Restaurant where eaten**  **(include address)** |
| Cucumbers (e.g., English, garden, mini, Persian) |  |  |  | |  | | |  | | | / / | |  | | / / | |  |
| Bell/sweet peppers (specify color/size) |  |  |  | |  | | |  | | | / / | |  | | / / | |  |
| Hot peppers/chilis (incl. salsa, and other dishes) |  |  |  | |  | | |  | | | / / | |  | | / / | |  |
| Asparagus |  |  |  | |  | | |  | | | / / | |  | | / / | |  |
| Celery |  |  |  | |  | | |  | | | / / | |  | | / / | |  |
| Carrots (specify baby or normal) |  |  |  | |  | | |  | | | / / | |  | | / / | |  |
| Radishes |  |  |  | |  | | |  | | | / / | |  | | / / | |  |
| Pea pods/snap peas |  |  |  | |  | | |  | | | / / | |  | | / / | |  |
| Onions (red/white/yellow) |  |  |  | |  | | |  | | | / / | |  | | / / | |  |
| Green onions/scallions |  |  |  | |  | | |  | | | / / | |  | | / / | |  |
| Broccoli |  |  |  | |  | | |  | | | / / | |  | | / / | |  |
| Cauliflower |  |  |  | |  | | |  | | | / / | |  | | / / | |  |
| Sprouts (e.g., alfalfa, bean, radish, clover) |  |  |  | |  | | |  | | | / / | |  | | / / | |  |
| Cilantro (incl. in fresh salsa and other dishes) |  |  |  | |  | | |  | | | / / | |  | | / / | |  |
| Other fresh herbs (e.g., basil, parsley, thyme, mint, sage) |  |  |  | |  | | |  | | | / / | |  | | / / | |  |
| Mushrooms (white, portabella, crimini) |  |  |  | |  | | |  | | | / / | |  | | / / | |  |
| Tofu/tempeh/seitan |  |  |  | |  | | |  | | | / / | |  | | / / | |  |
| Zucchini/summer squash (including spiralized) |  |  |  | |  | | |  | | | / / | |  | | / / | |  |
| **Item**  (vegetables cont.) | **Ate** | **Did not eat** | **May have eaten** | | **How prepared** | | | **Variety or brand** | | | **Date purchased** | | **Grocery store where purchased** | | **Date eaten** | | **Restaurant where eaten**  **(include address)** |
| Other vegetables |  |  |  | |  | | |  | | | / / | |  | | / / | |  |
| Pasta salad (ingredients/brand) |  |  |  | |  | | |  | | | / / | |  | | / / | |  |
| Potato salad (ingredients/brand) |  |  |  | |  | | |  | | | / / | |  | | / / | |  |
| **Item** | **Ate** | **Did not eat** | **May have eaten** | | **How prepared** | | | **Variety or brand** | | | **Date purchased** | | **Grocery store where purchased** | | **Date eaten** | | **Restaurant where eaten**  **(include address)** |
| **OTHER** |  | | | | | | | | | | | | | | | | |
| Nuts (e.g., almonds, pecans, walnuts, peanuts, cashews, other type)  -specify roasted, raw, in the shell |  |  |  | |  | | |  | | | / / | |  | | / / | |  |
| Hummus or tahini (specify) |  |  |  | |  | | |  | | | / / | |  | | / / | |  |
| Sesame seeds |  |  |  | |  | | |  | | | / / | |  | | / / | |  |
| Other seeds (e.g., flax, sunflower, chia, hemp) |  |  |  | |  | | |  | | | / / | |  | | / / | |  |
| Snacks such as soy nuts, corn nuts, chickpeas, wasabi peas |  |  |  | |  | | |  | | | / / | |  | | / / | |  |
| Salsa |  |  |  | |  | | |  | | | / / | |  | | / / | |  |
| Avocado (including guacamole) |  |  |  | |  | | |  | | | / / | |  | | / / | |  |
| Recently purchased spices (e.g., Black pepper, white pepper, paprika, oregano, cumin) |  |  |  | |  | | |  | | | / / | |  | | / / | |  |
| Spice rubs or blends |  |  |  | |  | | |  | | | / / | |  | | / / | |  |
| Peanut butter |  |  |  | |  | | |  | | | / / | |  | | / / | |  |
| **Item**  (Other cont.) | **Ate** | **Did not eat** | **May have eaten** | | **How prepared** | | | **Variety or brand** | | | **Date purchased** | | **Grocery store where purchased** | | **Date eaten** | | **Restaurant where eaten**  **(include address)** |
| Chocolate |  |  |  | |  | | |  | | | / / | |  | | / / | |  |
| Trail mix or granola |  |  |  | |  | | |  | | | / / | |  | | / / | |  |
| Chips, crackers, or other snack foods |  |  |  | |  | | |  | | | / / | |  | | / / | |  |
| Nutritional supplements, protein/whey powder, meal replacements |  |  |  | |  | | |  | | | / / | |  | | / / | |  |
| Anything I didn’t ask? |  |  |  | |  | | |  | | | / / | |  | | / / | |  |

| **If Adult Case:**  What is your occupation? _____________________________________  Name of employer? _________________________________________  Address/city of employer? ____________________________________  Work phone number_________________________________________  **If Child Case:**  Child’s school name/address:__________________________________  Parent 1 occupation: ________________________________________  Parent 2 occupation: ________________________________________  ***Updated 1/2018*** | **For Food Workers only:**  ***Salmonella* only:** People continue to shed  *Salmonella* in their stools after they recover.  You can return to work once you no longer have symptoms but it is essential that you have  excellent hand washing after going to the  bathroom and before handling foods to prevent transmitting *Salmonella* to patrons.  Statement read  ***E. coli* O157/STEC:**  Work restrictions/exclusions may apply to people  with *E. coli* O157/STEC infections who work in  food service. You will be contacted by an  epidemiologist if restrictions apply to you.  Statement read | **At the end of interview:**  Languages spoken at home:  English  Other(s): ___________  Hispanic or Non-Hispanic  *(please circle)*  Race: _______________  Specify: ____________ |
| --- | --- | --- |

***Read this if the case lived on, worked on, or visited a farm OR visited a petting zoo, educational exhibit, fair or other venue with animals. Do not read this if they work in a processing plant or went to a live animal market and have no other animal contact.***

**UMASH Study Reminder**

During the interview you indicated that you (or your child) had contact with farm animals prior to becoming ill. MDH is part of an agricultural safety and health center along with the University of Minnesota so someone from MDH will be calling you back to ask you a few additional questions about your (your child’s) farm animal exposure. Is there a specific day of the week or time of day that would be better to call you back? (If so, please indicate below).

Day of week (circle one): Sunday Monday Tuesday Wednesday Thursday Friday Saturday

Time of day: _________ 🞎 am 🞎 pm 🞎 No preference

*(for adult cases)* If you are not available to talk when we call, may we ask your spouse/partner these questions?

🞎 Yes 🞎 No Name:________________________________________________

Notes:______________________________________________________________________________________________________________________________________________________
