# Supplementary material for: Farm animal exposure setting impacts hemolytic uremic syndrome risk among Shiga toxin-producing Escherichia coli cases: Minnesota, 2010–2019
Source: Epidemiol Infect. 2024 May 16;152:e96. doi: 10.1017/S0950268824000773 (PMC11736442; doi:10.1017/S0950268824000773)
Supplement: Vachon et al. supplementary material 2 — Vachon et al. supplementary material [file S0950268824000773sup002.docx]

**Supplementary Material**

**Methods**

We imputed 40 datasets, 5 iterations each, to reduce the loss of efficiency to <1%, having confirmed that ≤40% of data was missing for any given variable [1]. The success of the imputation was determined by assessing diagnostic boxplots (continuous variables) and density plots (categorical variables).

**Council of State and Territorial Epidemiologists Case Definitions (Taken directly from the Centers from Disease Control and Prevention Website) [2-3]**

**Shiga Toxin-producing *Escherichia coli* (STEC)**

**2018 Case Definition**

## Background

Shiga toxin-producing *Escherichia coli* (STEC) are estimated to cause more than 265,000 illnesses each year in the United States. STEC can cause illness that ranges from mild diarrhea to bloody diarrhea and life-threatening hemolytic uremic syndrome (HUS). STEC are categorized into serogroups by their somatic O antigen. The STEC serogroup most commonly identified and associated with severe illness and hospitalization in the United States is *E. coli* O157; however, there are over 50 other serogroups that can also cause illness. The majority of infections are not reported to public health, because many individuals do not seek health care or are not tested. In recent years, the number of clinical laboratories that use tests that detect Shiga toxin or Shiga toxin genes has increased, resulting in increased detection of both O157 and non-O157 STEC infections.

Ongoing surveillance of STEC infections is essential to detect and control outbreaks, to determine public health priorities, to monitor trends in illness, and to assess effectiveness of public health interventions. Methods for surveillance must keep pace with changing laboratory diagnostic methods.

## Clinical Criteria

An infection of variable severity characterized by diarrhea (often bloody) and/or abdominal cramps. Illness may be complicated by HUS (note that some clinicians still use the term thrombotic thrombocytopenic purpura [TTP] for adults with post-diarrheal HUS).

## Laboratory Criteria For Diagnosis

*Confirmatory laboratory evidence*

- Isolation of *E. coli* O157:H7 from a clinical specimen **OR**
- Isolation of *E. coli* from a clinical specimen with detection of Shiga toxin or Shiga toxin genes.

*Supportive laboratory evidence*

- Isolation of *E. coli* O157 from a clinical specimen without confirmation of H antigen, detection of Shiga toxin, or detection of Shiga toxin genes, **OR**
- Identification of an elevated antibody titer against a known Shiga toxin-producing serogroup of *E. coli*, **OR**
- Detection of Shiga toxin or Shiga toxin genes in a clinical specimen using a culture-independent diagnostic test (CIDT) and no known isolation of *Shigella* from a clinical specimen. **OR**
- Detection of *E. coli* O157 or STEC/ Enterohemorrhagic *E. coli* (EHEC) in a clinical specimen using a CIDT.

## Epidemiologic Linkage

A clinically compatible illness in a person that is epidemiologically linked to a confirmed or probable case with laboratory evidence **OR**

A clinically compatible illness in a person that is a member of a risk group as defined by public health authorities during an outbreak.

## Criteria to Distinguish a New Case from an Existing Case

A new case should be created when a positive laboratory result is received more than 180 days after the most recent positive laboratory result associated with a previously reported case in the same individual. (See formula referenced in Appendix B of the 2017 CSTE Position Statement [17-ID-10] for details on time period calculation, hierarchy of dates and interpretation). **OR**

When two or more different serogroups/serotypes are identified in one or more specimens from the same individual, each serogroup/serotype should be reported as a separate case.

## Case Classification

### Suspected

- Identification of an elevated antibody titer against a known Shiga toxin-producing serogroup of E.coli in a person with no known clinical compatibility, **OR**
- Detection of Shiga toxin or Shiga toxin genes in a clinical specimen using a CIDT and no known isolation of Shigella from a clinical specimen in a person with no known clinical compatibility, **OR**
- Detection of E. coli O157 or STEC/EHEC in a clinical specimen using a CIDT in a person with no known clinical compatibility, **OR**
- A person with a diagnosis of post-diarrheal HUS/TTP (see HUS case definition).

### Probable

- A person with isolation of *E. coli* O157 from a clinical specimen without confirmation of H antigen, detection of Shiga toxin or detection of Shiga toxin genes, **OR**
- A clinically compatible illness in a person with identification of an elevated antibody titer against a known Shiga toxin-producing serogroup of *E. coli*, **OR**
- A clinically compatible illness in a person with detection of Shiga toxin or Shiga toxin genes in a clinical specimen using a CIDT and no known isolation of *Shigella* from a clinical specimen, **OR**
- A clinically compatible illness in a person with detection of E. coli O157 or STEC/EHEC from a clinical specimen using a CIDT, **OR**
- A clinically compatible illness in a person that is epidemiologically linked to a confirmed or probable case with laboratory evidence, **OR**
- A clinically compatible illness in a person that is a member of a risk group as defined by public health authorities during an outbreak.

### Confirmed

- A person that meets the confirmatory laboratory criteria for diagnosis.

# Shiga Toxin-producing *Escherichia coli* (STEC)

# 2014 Case Definition

## Background

Shiga-toxin producing *Escherichia coli* (STEC) can cause illness that ranges from mild diarrhea to bloody diarrhea, and life-threatening hemolytic uremic syndrome (HUS). STEC are categorized into serogroups by their somatic O antigen. The STEC serogroup most commonly identified and associated with severe illness in the United States is *E. coli* O157; however, there are over 50 other serogroups that can cause illness.

## Clinical Description

An infection of variable severity characterized by diarrhea (often bloody) and abdominal cramps. Illness may be complicated by Hemolytic Uremic Syndrome (HUS). (Note, some clinicians still use the term thrombotic thrombocytopenic purpura [TTP] for adults with post-diarrheal HUS.); asymptomatic infections also may occur, and the organism may rarely cause extraintestinal infections.

## Laboratory Criteria For Diagnosis

Laboratory confirmed

- Isolation of STEC from a clinical specimen. *Escherichia coli* O157 isolates that produce the H7 antigen may be assumed to be Shiga toxin-producing. For all other *E. coli* isolates, Shiga toxin production or the presence of Shiga toxin genes must be determined to be considered STEC.
- Both asymptomatic infections and infections at sites other than the gastrointestinal tract, if laboratory confirmed, are considered confirmed cases that should be reported.

Supportive laboratory results

- A case with isolation of *E. coli* O157 from a clinical specimen, without confirmation of H antigen or Shiga toxin production
- Identification of an elevated antibody titer to a known STEC serotype from a clinically compatible case
- Identification of Shiga toxin in a specimen from a clinically compatible case without the isolation of STEC

## Epidemiologic Linkage

A clinically compatible case that is epidemiologically linked to a confirmed or probable case.

## Case Classification

### Suspected

- A case of postdiarrheal HUS (see HUS case definition) **OR**
- Identification of Shiga toxin in a specimen from a clinically compatible case without the isolation of STEC

### Probable

- A case with isolation of *E. coli* O157 from a clinical specimen, without confirmation of H antigen or Shiga toxin production. **OR**
- A clinically compatible case who is a contact of an STEC case or is a member of a defined risk group during an outbreak. **OR**
- Identification of an elevated antibody titer to a known STEC serotype from a clinically compatible case

### Confirmed

- A case that meets the confirmed laboratory criteria for diagnosis. When available, O and H antigen serotype characterization should be reported.

# Hemolytic Uremic Syndrome, Post-diarrheal (HUS) 1996 Case Definition

## Clinical Description

Hemolytic uremic syndrome (HUS) is characterized by the acute onset of microangiopathic hemolytic anemia, renal injury, and low platelet count. Thrombotic thrombocytopenic purpura (TTP) also is characterized by these features but can include central nervous system (CNS) involvement and fever and may have a more gradual onset. Most cases of HUS (but few cases of TTP) occur after an acute gastrointestinal illness (usually diarrheal).

## Laboratory Criteria For Diagnosis

The following are both present at some time during the illness:

- Anemia (acute onset) with microangiopathic changes (i.e., schistocytes, burr cells, or helmet cells) on peripheral blood smear, **AND**
- Renal injury (acute onset) evidenced by either hematuria, proteinuria, or elevated creatinine level (i.e., greater than or equal to 1.0 mg/dL in a child aged less than 13 years or greater than or equal to 1.5 mg/dL in a person aged greater than or equal to 13 years, or greater than or equal to 50% increase over baseline)

Note: A low platelet count can usually, but not always, be detected early in the illness, but it may then become normal or even high. If a platelet count obtained within 7 days after onset of the acute gastrointestinal illness is not less than 150,000/mm^3^, other diagnoses should be considered.

## Case Classification

### Probable

- An acute illness diagnosed as HUS or TTP that meets the laboratory criteria in a patient who does not have a clear history of acute or bloody diarrhea in preceding 3 weeks, **OR**
- An acute illness diagnosed as HUS or TTP, that a) has onset within 3 weeks after onset of an acute or bloody diarrhea and b) meets the laboratory criteria except that microangiopathic changes are not confirmed

### Confirmed

An acute illness diagnosed as HUS or TTP that both meets the laboratory criteria and began within 3 weeks after onset of an episode of acute or bloody diarrhea

## Comments

Some investigators consider HUS and TTP to be part of a continuum of disease. Therefore, criteria for diagnosing TTP on the basis of CNS involvement and fever are not provided because cases diagnosed clinically as post-diarrheal TTP also should meet the criteria for HUS. These cases are reported as post-diarrheal HUS. Most diarrhea-associated HUS is caused by Shiga toxin-producing *Escherichia coli*, most commonly *E. coli* O157. If a patient meets the case definition for both shiga toxin-producing *E. coli* (STEC) and HUS, the case should be reported for each of the conditions.

The 1996 case definition appearing on this page was re-published in the 2009 CSTE position statement 09-ID-37. Thus, the 1996 and 2010 versions of the case definition are identical.

**References Cited**

1. Schomaker, M., Heumann, C. (2018). Bootstrap inference when using multiple imputation. *Stat Med,37*:2252–66.
2. Centers for Disease Control and Prevention. (2021). National Notifiable Disease Surveillance System (NNDSS) Shiga toxin-producing *Escherichia coli* (STEC). Available at <https://ndc.services.cdc.gov/case-definitions/shiga-toxin-producing-escherichia-coli-2018/> (accessed 2 October 2022).
3. Centers for Disease Control and Prevention. (2021). National Notifiable Diseases Surveillance System (NNDSS). Hemolytic Uremic Syndrome, Post-diarrheal (HUS). Available at <https://ndc.services.cdc.gov/case-definitions/hemolytic-uremic-syndrome-post-diarrheal/> (accessed 2 October 2022).
